# The odds and costs of ambulance attendances associated with heatwave severity in older adults of queensland, Australia

**DOI:** 10.1007/s00484-025-02981-w

**Published:** 2025-07-08

**Authors:** Zhiwei Xu, Shannon Rutherford, Son Nghiem, Hannah M. Mason, Blesson Mathew Varghese, Jemma C. King, Amy E. Peden, Kerrianne Watt, Emma Bosley, Richard C. Franklin

**Affiliations:** 1 School of Medicine and Dentistry, Griffith University, Gold Coast, 4222 QLD Australia; 2 Cities Research Institute, Griffith University, Gold Coast, Australia; 3 Department of Health, Economics, Wellbeing and Society, National Centre for Epidemiology and Population Health (NCEPH), College of Health and Medicine, The Australian National University, Canberra, Australia; 4 College of Public Health, Medical and Veterinary Sciences, James Cook University, Townsville, QLD 4811 Australia; 5 School of Public Health, the University of Adelaide, Adelaide, Australia; 6 School of Population Health, Faculty of Medicine and Health, University of New South Wales, Sydney, NSW 2052 Australia; 7 Information Support, Research & Evaluation, Queensland Ambulance Service, Brisbane, QLD 4031 Australia; 8 School of Clinical Sciences, Faculty of Health, Queensland University of Technology, Brisbane, Australia; 9 School of Public Health and Social Work, Faculty of Health, Queensland University of Technology, Brisbane, Australia

**Keywords:** Heatwaves, Heat early warning system, Health costs, Ambulance, Elderly

## Abstract

Ambulance attendances have been increasingly reported to be a sensitive indicator of increased health service utilisations during heatwaves. This study estimated the odds and costs of ambulance attendances associated with heatwave severity (low-intensity, severe, and extreme) in older adults (≥ 65 years) from 1st January 2010 to 31st December 2018 in the State of Queensland, Australia. The findings showed that low-intensity, severe, and extreme heatwaves were associated with 1.4% (95% confidence interval: 0.3%, 2.5%), 4.1% (2.1%, 6.1%), and 9.7% (7.3%, 12.0%) increased odds of ambulance attendances for older adults, respectively. The increased odds of ambulance attendances elevated with age during extreme heatwaves (peaking at 11.7% for ≥ 85 years). Heatwaves were associated with additional ambulance attendance costs of $237,141 per year for older adults in Queensland, with 62.2% and 31.7% of these costs from low-intensity and severe heatwaves, respectively. These findings call for research to address critical knowledge gaps around appropriate early warning system notifications for low-intensity and severe heatwaves, given their evidenced impacts on individuals and emergency health systems.

## Introduction

Prolonged days of extreme heat (i.e., heatwaves) are detrimental to health and well-being, causing heat-related illnesses (e.g., heat stroke) (Sorensen and Hess 2022) and/or triggering the exacerbation of pre-existing chronic diseases (e.g., cardiovascular diseases) (Ebi et al. 2021), increasing the demand for health service utilisations (Mason et al. 2022). Older adults (≥ 65 years) are particularly susceptible to the health impacts of heatwaves, due to their age-related decline in thermoregulatory capacity (Meade et al. 2020) and their higher prevalence of chronic diseases (Xu et al. 2024).

Exposure to heatwaves is associated with an increased likelihood of hospitalisations and emergency department visits (PoshtMashhadi et al. 2025). Evidence also suggests that, compared to hospitalisations and emergency department visits, ambulance attendances may be a more sensitive indicator of increased demand for health service utilisation during heatwave days (Xu et al. 2018). This evidence is useful for heat early warning systems because these systems often include a component of real-time surveillance for the health impacts of heatwaves (Matthies et al. 2008). Aside from statistics on heatwave-related health impacts, data on the additional healthcare costs incurred during heatwaves are equally important to inform decision-making on resource allocation for heat early warning systems. This is because such decisions often (if not always) require consideration of both costs and benefits. An evidence-based heat early warning system, supported by adequate resources, is more likely to reduce heatwave-related health consequences than one that is poorly designed and implemented. Although there is published evidence on the additional costs of hospitalisations and emergency department visits during heatwaves (Wondmagegn et al. 2019), evidence on the additional costs of ambulance attendances during heatwaves remains scarce, both in Australia (Campbell et al. 2023) and globally.

In this study, we conducted a statewide analysis to (1) estimate the odds of ambulance attendances associated with different severities of heatwaves in older adults in the State of Queensland, Australia; (2) estimate the heatwave-related costs of ambulance attendances; and (3) further estimate the odds and costs by their age, sex, and rurality of the ambulance attendance location.

## Materials and methods

### Study site

The study site was the State of Queensland, Australia. Queensland is the 3rd most populous state in Australia, with a resident population of 5.2 million people in 2021 (Australian Bureau of Statistics 2021). Queensland is served by a single, government-funded emergency ambulance service (i.e., the Queensland Ambulance Service [QAS]). The QAS provides prehospital attendance to individuals in need and the presence of this service across the entire state enables emergency response irrespective of location and rurality of residence.

### Data collection

Data on emergency ambulance calls resulting in ambulance attendance (hereafter referred to as “ambulance attendances” for simplicity) in older adults (≥ 65 years) from 1 st January 2010 to 31 st December 2018 were obtained from the QAS. The data included the date of the ambulance attendance, the age and sex of each individual, and the postcode and rurality status of the ambulance attendance location. Age was categorised into three groups: 65–74, 75–84, and ≥ 85 years. The Australian Statistical Geography Standard (ASGS) Remoteness Structure categorises rurality status into five groups: major cities, inner regional, outer regional, remote, and very remote (Australian Bureau of Statistics 2011). In this study, because of the limited number of ambulance attendances in ‘remote’ and ‘very remote’ suburbs, ‘remote’ and ‘very remote’ were collapsed into one category called ‘remote or very remote’ to facilitate analysis.

There is no international consensus on how to define a heatwave. The Australian Bureau of Meteorology (BoM) defines a heatwave as a period when the maximum and minimum temperatures are unusually hot over three days, compared to the local climate and past weather. The Australian BoM uses the excess heat factor (EHF) (Nairn and Fawcett 2015) to categorise heatwaves into three severities: low-intensity, severe, and extreme heatwaves. As the Australian BoM issues heatwave early warnings based on EHF in Australia, EHF was used to define heatwave severities in our study to generate policy-relevant evidence. Following our previous practice (Xu et al. 2023a), publicly available spatially refined gridded data on daily maximum and minimum temperatures (5 km*5km) from 1 st January 1971 to 31 st December 2018 were obtained (SILO 2023), and converted into postcode-level maximum and minimum temperature data. The postcode-level maximum and minimum temperature data were utilised to further calculate postcode-level EHF and identify the days from 2010 to 2018 when there were low-intensity, severe, or extreme heatwaves.

### Data analysis

A two-stage analysis was conducted to estimate the odds and costs of ambulance attendances associated with heatwave severities.

Stage 1: the association between heatwaves and the odds of ambulance attendances was quantified using a time-stratified case-crossover design. Case-crossover design has been widely used to quantify the association between short-term exposure to an environmental hazard and the likelihood of a health event (Carracedo-Martínez et al. 2010). In this study, the heatwave exposure on the day when an ambulance attendance occurred at a postcode (case days) was compared with the heatwave exposure on the same day of other weeks in the same month at the same postcode (control days). Conditional logistic regression was used to estimate the odds ratios (ORs) and 95% confidence intervals (CIs) of ambulance attendances associated with heatwaves in Queensland. The published evidence suggested that the impacts of short-term heatwave exposure on ambulance attendances can last beyond the concurrent day of exposure and several days after exposure (Gronlund et al. 2014; Rizmie et al. 2022). Our exploratory analysis showed that the increased odds of ambulance attendances became statistically non-significant on the 7th day after heatwave exposure. Hence, we estimated the ORs and 95% CIs across the concurrent day of exposure and six days after exposure (i.e., lag 0–6 days). Following our previous practice (Tao et al. 2022), we used random-effects meta-analyses to pool the ORs and 95% CIs across the lag days to retrieve a pooled effect estimate. Aside from the analysis for the whole population of older adults, we further conducted analyses by age, sex, and rurality.

Stage 2: based on the pooled effect estimates yielded in stage 1, we estimated the costs of ambulance attendances associated with heatwaves using the following formula:

$$\mathrm{Heat\;wave}-\mathrm{related\;ambulance\;attendance\;costs}=\left(\mathrm{OR}-1\right)\ast\mathrm A\ast\mathrm C$$

Where OR represents the odds ratio of ambulance attendances during heatwave days (e.g., low-intensity heatwave days vs. no heatwave days), ‘A’ represents the total observed number of ambulance attendances during heatwave days, and ‘C’ represents the average cost of an ambulance attendance in Queensland. Data on the average cost of an ambulance attendance was obtained from the Queensland Ambulance Service Public Performance Indicators from 2010 to 2024 (Queensland Ambulance Service 2024). On average, it cost AU$700.8 per ambulance attendance in Queensland. However, after adjusting for inflation using the Consumer Price Index (Australian Bureau of Statistics 2024), the average cost increased to AU$747.64 (2024 price). For simplicity, hereinafter, ‘$’ refers to ‘AU$, 2024 price’. In Queensland, the Queensland Ambulance Service is fully funded by the state government, and permanent residents receive ambulance services without direct charges.

Due to the absence of additional linked data (e.g., emergency department presentations, hospitalisations), we were unable to estimate other costs associated with heatwaves (e.g., hospital length of stays, pharmaceutical, pathology) and indirect costs (e.g., work absenteeism, disability, or premature deaths).

All data analyses were conducted in R, with the ‘metafor’ package being used to conduct the meta-analyses.

### Ethics

Ethics approval was granted by the Children’s Health Queensland Hospital and Health Service Human Research Ethics Committee (HREC/21/QCHQ/72044) before the ambulance data collection. After obtaining the ethics approval, we further obtained approval from the Queensland Ambulance Service for accessing the ambulance data (QAS114).

## Results

There were 2,210,036 recorded ambulance attendances in older adults from 2010 to 2018 (Table 1). 71.3% of these attendances occurred in persons under the age of 85 (i.e., 65–74, 75–84). There were more ambulance attendances for males than females (52.5% vs. 47.5%). Unsurprisingly, a substantial proportion of ambulance attendances occurred in major cities (60.2%). Across all postcodes, there was a yearly average of 80 (range: 50–103) low-intensity heatwave days, 23 (range: 8–38) severe heatwave days, and 3 (range: 0–9) extreme heatwave days.

**Table 1:** Ambulance attendances in people aged ≥ 65 years in queensland, australia, from 1 st January 2010 to 31 st December 2018, by age, sex, and rurality

	Number (%*)	Number during heatwave days (%*)
Overall	2,210,036	136,972
Age (years)		
65–74	744,019 (33.7)	46,499 (33.9)
75–84	830,326 (37.6)	51,155 (37.3)
≥ 85	635,691 (28.8)	39,318 (28.7)
Sex		
Male	1,158,365 (52.5)	65,038 (47.5)
Female	1,049,633 (47.5)	71,759 (52.5)
Rurality		
Major cities	1,329,184 (60.1)	87,707 (64.1)
Inner regional	533,172 (24.1)	30,499 (22.3)
Outer regional	309,218 (14.0)	16,515 (12.1)
Remote or very remote	35,052 (1.6)	2,033 (1.5)

*The per cent is the column per cent, not the row per cent

**Fig. 1:**
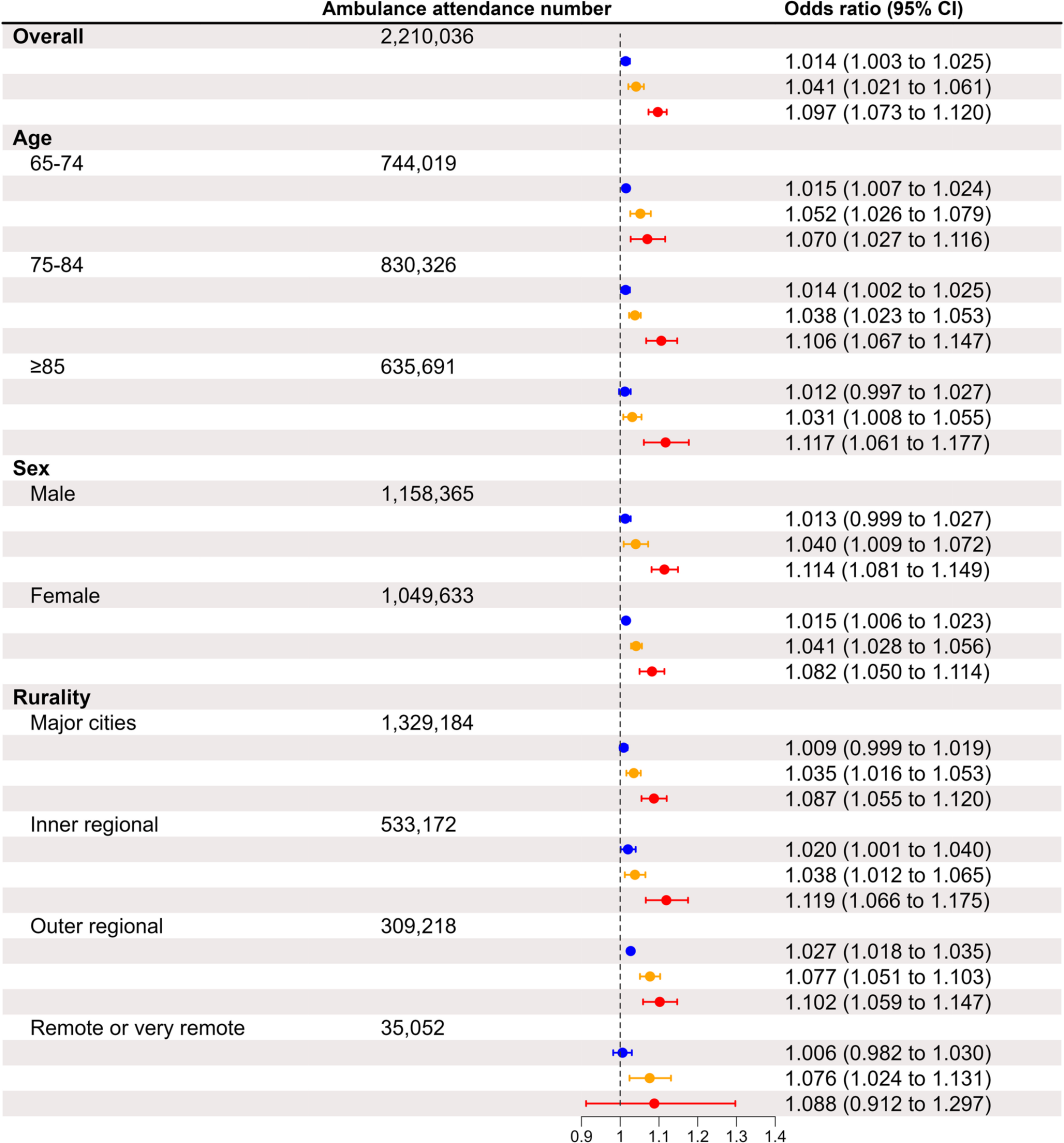
Increased odds of ambulance attendances during heatwaves of different severities in older adults in Queensland and by age, sex, and rurality of the ambulance attendance location. Blue, orange, and red colours represent the odds ratios and their 95% confidence intervals of ambulance attendances during low-intensity, severe, and extreme heatwaves, respectively

The odds of ambulance attendances associated with heatwaves increased with heatwave intensity (Fig. 1). Low-intensity, severe, and extreme heatwaves were associated with 1.4% (95% confidence interval [CI]: 0.3%, 2.5%), 4.1% (2.1%, 6.1%), and 9.7% (7.3%, 12.0%) increased odds of ambulance attendances, respectively. Across all age, sex, and rurality groups, the odds of ambulance attendances associated with heatwaves increased with heatwave severity.

During extreme heatwaves, the odds of ambulance attendances associated with heatwaves elevated with age, with those aged 65–74, 75–84, and ≥ 85 years experiencing 7.0% (95% CI: 2.7%, 11.6%), 10.6% (6.7%, 14.7%), and 11.7% (6.1%, 17.7%) increased odds, respectively (Fig. 1). However, this age-related pattern was reversed during low-intensity or severe heatwaves.

Throughout the 9-year study period, the cost associated with ambulance attendances to older adults due to heatwaves in Queensland was $2,134,273, of which 62.2% ($1,327,454) was from low-intensity heatwaves, 31.7% ($677,121) from severe heatwaves, and 6.1% ($129,698) from extreme heatwaves (Table 2). The highest cost (805,219) was among those aged 65–74 years, and the lowest cost (532,317) was among those aged ≥ 85 years (Table 2). The costs of ambulance attendances associated with heatwaves were higher for females (1,169,930) than for males (972,724), primarily due to the higher odds of ambulance attendance in females in low-intensity heatwaves, and greater frequency of low-intensity heatwaves (hence higher costs associated with low-intensity heatwaves in females [$755,845] compared to males [$577,606]).

**Table 2:** Costs of ambulance attendances associated with heatwaves in queensland, from 1 st January 2010 to 31 st December 2018

	Heatwave severity	Costs	95% confidence interval	
			Lower limit	Upper limit
Overall		**$2**,**134**,**273**		
	Low	$1,327,454	$1,300,952	$1,353,956
	Severe	$677,121	$668,441	$685,801
	Extreme	$129,698	$128,858	$130,539
Aged 65–74 years		**$805**,**219**		
	Low	$480,263	$473,102	$487,424
	Severe	$295,381	$291,486	$299,276
	Extreme	$29,575	$29,078	$30,072
Aged 75–84 years		**$784**,**301**		
	Low	$495,825	$485,364	$506,286
	Severe	$236,443	$234,006	$238,881
	Extreme	$52,033	$51,517	$52,548
Aged ≥ 85 years		**$532**,**317**		
	Low	$337,426	$326,986	$347,865
	Severe	$145,980	$142,164	$149,796
	Extreme	$48,911	$48,273	$49,549
Male		**$972**,**724**		
	Low	$577,606	$561,070	$594,143
	Severe	$319,474	$312,935	$326,013
	Extreme	$75,644	$75,052	$76,237
Female		**$1**,**169**,**930**		
	Low	$755,845	$745,059	$766,631
	Severe	$358,776	$355,604	$361,948
	Extreme	$55,309	$54,752	$55,865
Major cities		**$994**,**168**		
	Low	$559,434	$543,994	$574,874
	Severe	$373,459	$368,246	$378,672
	Extreme	$61,275	$60,672	$61,879
Inner regional		**$601**,**332**		
	Low	$425,661	$414,789	$436,532
	Severe	$143,875	$141,270	$146,480
	Extreme	$31,796	$31,409	$32,182
Outer regional		**$486**,**293**		
	Low	$311,121	$308,609	$313,633
	Severe	$140,673	$139,418	$141,928
	Extreme	$34,499	$34,111	$34,888
Remote or very remote		**$28**,**916**		
	Low	$8,592	$7,689	$9,495
	Severe	$18,510	$18,176	$18,844
	Extreme	$1,814	$1,710	$1,917

## Discussion

This study has observed several useful findings: (1) the odds of ambulance attendances associated with heatwaves increased with heatwave severity in older adults and across age, sex, and rurality subgroups; (2) during extreme heatwaves, the odds of ambulance attendances associated with heatwaves elevated with age, but this age-related pattern was reversed during low-intensity or severe heatwaves; (3) heatwaves were associated with additional ambulance attendance costs of $237,141 per year in older adults in Queensland, with 93.9% of the additional costs from low-intensity and severe heatwaves.

The increases in the odds of ambulance attendances during low-intensity (1.4%), severe (4.1%), and extreme heatwaves (9.7%) observed in older adults were smaller than those observed in the State of Tasmania, Australia (4%, 13%, and 47%, respectively) (Campbell et al. 2021), and smaller than those reported in a systematic review paper for the general population in Australia (6%, 7%, and 18%, respectively) (Xu et al. 2023b). The discrepancies might have been due to the different lag periods used in our study and the previous studies. For instance, in the Tasmania study, Campbell et al. examined the impact that occurred on the concurrent day of heatwave exposure (i.e., lag 0), and our study estimated the average impact from the concurrent day of heatwave exposure to six days after exposure (i.e., lag 0–6 days). Because the greatest heatwave impact often occurs on the concurrent day of exposure, using the average impact over a longer lag could have yielded a smaller effect estimate. Aside from the different lag periods used, the finding may also have been because older adults in Queensland have higher access to cooling measures than those in Tasmania. For instance, a survey conducted by the Australian Bureau of Statistics showed that the prevalence of air conditioning in Queensland was higher than that in Tasmania in 2014 (the midpoint of our study period) (74% vs. 52%) (Australian Bureau of Statistics 2014). Although the increase in the odds of ambulance attendances associated with heatwaves in Queensland was lower than that in Tasmania, the yearly costs of ambulance attendances associated with heatwaves in Queensland ($237,141) were higher than those in Tasmania ($57,147) (Campbell et al. 2023), quite likely due to the much larger population in Queensland than in Tasmania (5.2 million vs. 0.56 million) (Australian Bureau of Statistics 2021).

As people age, they tend to have a higher heat susceptibility due to age-related decline in thermoregulatory function (Meade et al. 2020) and a greater prevalence of chronic diseases (Xu et al. 2024). Hence, we hypothesised that the increased odds of ambulance attendances associated with heatwaves would elevate with age, but we only found this pattern during extreme heatwaves, and not during low-intensity or severe heatwaves. Two reasons may explain this finding: (1) generally, persons aged 85 years or above are less active outside during low-intensity and severe heatwaves compared to their younger counterparts, which may result in less exposure to these heatwaves; (2) persons aged 85 years or above tend to take more protective measures (i.e., wearing cooler clothing, using air-conditioning) even during low-intensity or severe heatwaves (Harvey et al. 2024). Furthermore, in Australians aged 85 years or above, a lower proportion live in the community compared to their younger counterparts, with more residing in residential aged care facilities and nursing homes. During low-intensity and severe heatwaves, staff in these facilities may turn on air-conditioning to protect older adults from the impacts of heatwaves and ensure older adults are being cared for.

We found that 62.2% and 31.7% of heatwave-related ambulance costs were attributable to low-intensity and severe heatwaves respectively. This finding echoed our previous finding in the three biggest Australian cities that 64.9% and 30.4% of heatwave-related deaths were attributable to low-intensity and severe heatwaves, respectively (Xu et al. 2023a). Although the odds of ambulance attendances associated with each low-intensity heatwave day were not as high as those associated with each extreme heatwave day, low-intensity heatwave days (yearly average number across Queensland = 80) were far more common than extreme heatwave days (yearly average number across Queensland = 3), hence making their attributable burden much higher. In Queensland, the heat early warning system (called Queensland Heatwave Management Sub-plan) was first developed in 2015 and has been updated periodically (Queensland Health 2023). Since 2023, this early warning system has been triggered primarily during extreme heatwaves due to warning fatigue concerns. Research is warranted to address critical knowledge gaps around the varying degrees of heat health risk across and within subpopulations (Xu et al. 2024), because it would enable the heat early warning system to provide nuanced targeting of those most at risk during heatwaves of different severities. For instance, it may be worthwhile to consider issuing alerts to those most at risk on low-intensity and severe heatwave days and issuing warnings to those most at risk and the general public on extreme heatwave days. The authors (e.g., SR and RF) have been providing consultancy support to Queensland Health for updating the Queensland Heatwave Management Sub-plan. The findings of this study are expected to be shared with Queensland Health for consideration in the next iteration of the Sub-plan, as well as the QAS Heatwave Management Plan.

The Sustainable Development Goals (SDGs), adopted by the United Nations, are a global call to action for all countries to end poverty, protect the planet, and ensure that all people enjoy peace and prosperity by 2030. SDG 13 focuses on taking urgent action to combat climate change and its impacts - one of which is the increasing frequency of extreme weather events, such as heatwaves. A recommended strategy to address the impacts of heatwaves is the development of evidence-based heat early warning systems. This study, alongside other research on heat early warnings in Australia and beyond, is expected to inform the development and refinement of such systems, thereby contributing to the achievement of SDG 13.

The present study extends knowledge about the service demand and costs of ambulance attendances associated with heatwave severity. In this epidemiological study, although we used the best available population-level ambulance data in Queensland, this study has three main limitations: First, as the ambulance data were not linked to hospitalisation data, we were unable to estimate other costs associated with heatwaves such as hospitalisations and productivity loss. Second, because the ambulance attendance data did not include definitive diagnoses, we were unable to estimate the association between heatwaves and cause-specific ambulance attendances. Definitive diagnostic data on causes of illness/injury such as that in hospitalisation data (e.g., diagnoses coded in the International Classification of Diseases 10th Revision [ICD-10]) would facilitate the estimation of heatwave-related costs due to biologically plausible heat-sensitive diseases including cardiovascular diseases (Cheng et al. 2019), diabetes (Gao et al. 2022), respiratory diseases (Cheng et al. 2019), kidney diseases (Liu et al. 2021a), and mental disorders (Liu et al. 2021b). Third, the ambulance attendance cost data used in this study may have not accurately reflected resource consumption. The number and type of ambulance units attending the incident were not reflected here, as data were deduplicated to include one person record per incident. Some incidents had multiple patients and some incidents received multiple ambulance units in response. A three-tiered response model has been utilised by the QAS, including advanced care paramedics [ACPs], critical care paramedics [CCPs], and high acuity response unit [HARU], each with varying qualification/training requirements, scope of practice, and remuneration. Additionally, some incidents require an aeromedical response (e.g., helicopter, or fixed wing). The data used in this study also did not reflect the total volume of work (i.e., how long the dispatcher spent on each call, how long paramedics were present at the scene of the incident, and how long paramedics were present in the emergency department). This total volume of work may change during a heatwave as a function of increased demand for ambulance attendances and other emergency health services. Hence, it is likely that the cost estimations of our study represent an underestimate of the true magnitude and cost of ambulance attendances associated with heatwaves in Queensland.

## Conclusion

In Queensland, heatwaves are associated with older adults’ increased attendances to ambulance service, leading to $237,141 in additional costs of ambulance attendances each year. Although the odds of ambulance attendances associated with heatwaves increase with heatwave severity, 93.9% of heatwave-related ambulance costs are from low-intensity and severe heatwaves because these heatwaves occur more frequently. To enable the current population-based heat early warning systems to prevent adverse health consequences during low-intensity and severe heatwaves, research is warranted to understand the varying degrees of heat-health risk across and within Australian subpopulations.

## Data Availability

Ambulance attendance data used in this study were provided by the Queensland Ambulance Service (https://www.ambulance.qld.gov.au/). Access to this ambulance data requires ethics approval.
